# Supplementary material for: Parametric Copula-GP model for analyzing multidimensional neuronal and behavioral relationships
Source: PLoS Comput Biol. 2022 Jan 28;18(1):e1009799. doi: 10.1371/journal.pcbi.1009799 (PMC8827448; doi:10.1371/journal.pcbi.1009799)
Supplement: S1 Text — Include Bayesian model selection details and mixture model construction strategies. (PDF) [file pcbi.1009799.s001.pdf]

S1 Text: Supplementary methods for "Parametric Copula-GP model for analyzing multidimensional neuronal and behavioral relationships"

January 10, 2022

Contents

A Bayesian model selection **1**

B Model selection algorithms **1**

C Effective learning rates for different families **3**

A Bayesian model selection

In model selection, we are aiming to construct a model with the lowest possible WAIC. Since our copula probability densities are continuous, their values can exceed 1 and the resulting WAIC is typically negative. Zero WAIC corresponds to the Independence model (pdf = 1 on the whole unit square). We also set up a tolerance ($\mathrm{WAIC}_{tol} = 0.005$), and models with $\mathrm{WAIC} \in [-\mathrm{WAIC}_{tol}, \mathrm{WAIC}_{tol}]$ are considered indistinguishable from the independence model.

Since the total number of combinations of 10 copula elements (Fig 5) is large, exhaustive search for the optimal model is not feasible. In our framework, we propose two model algorithms for constructing close-to-optimal copula mixtures: *greedy* and *heuristic*.

B Model selection algorithms

The greedy algorithm (Algorithm 1) starts by comparing WAIC of all possible single-copula models (from Table 1, in all rotations) and selecting the model with the lowest WAIC. After that, we add one more copula (from another family or in another rotation) to the first selected copula, and prepend the element that yields the lowest WAIC of the mixture. We repeat the process until the WAIC stops decreasing. After the best model is selected, we remove the inessential elements using the `reduce(.)` function. This function removes those elements which have an average concentration of $< 10\%$ everywhere on $x \in [0, 1]$. This step is added to improve the interpretability of the models and computation time for entropy estimation (at a small accuracy cost) and can, in principle, be omitted. The selection process takes approximately (30-60) minutes$\times$number of copulas in the correct mixture on a RTX 2080Ti , but terminates in less than 1 minute for independent variables.

Algorithm 1: Greedy algorithm for copula mixture selection

```
M, M_old ← [ ], [ ];
S_c ← [Independence, Gauss, Frank, 4 × Clayton, 4 × Gumbel];
                                        // 4× includes all rotations
// while every update of the model yields a new best
while WAIC(M) ≤ WAIC(M_old) and size(S_c) > 0 do
    M_old ← M ;
    select c from S_c such that WAIC(prepend(c, M)) is minimal;
    M ← prepend(c, M) ;
    remove c from S_c;
end
M_best ← reduce(M_old);
return M_best;
```

Algorithm 2: Heuristic algorithm for copula mixture selection

```
G ← [Gauss];
if WAIC(G) > −waic_tol then
    return [Independence];
end
M_Cl ← [Independence, Gauss, 4 × Clayton];
M_Gu ← [Independence, Gauss, 4 × Gumbel];
M_best, M_worst ← (M_Cl, M_Gu) sorted by WAIC;
if WAIC(G) < WAIC(M_best) then
    return G;
end
for i ← 3 . . . size(M_best) do
    M ← M_best with i-th element replaced by M_worst[i];
    M_best ← M if WAIC(M) < WAIC(M_best);
end
M_best ← reduce(M_best);
if Gauss ∈ M_best then
    M ← M_best with Gauss replaced by Frank;
    M_best ← M if WAIC(M) < WAIC(M_best);
end
// Gauss often gets confused with pairs of e.g.
    Clayton 0° + Gumbel 0°
if size(M_best) > 1 then
    for i ← 1 . . . (size(M_best) − 1) do
        for j ← (i + 1) . . . size(M_best) do
            M ← M_best with i-th and j-th elements removed;
            M ← prepend(Gauss, M);
            if WAIC(M) < WAIC(M_best) then
                M_best ← M ;
                break;
            end
        end
    end
end
M_best ← reduce(M_best);
return M_best;
```

The greedy algorithm can be improved by adding model reduction after each attempt to add an element. In this case, the number of elements can increase and decrease multiple times during the model selection process, which also must be terminated if the algorithm returns to the previously observed solution. Even though it complicates the algorithm, it reduces the maximal execution time (observed on the real neuronal data) from ∼90 minutes down to ∼40 minutes.

The heuristic algorithm focuses on the tail dependencies (Algorithm 2). First, we try a single Gaussian copula. If variables are not independent, we next compare 2 combinations of 6 elements, which are organized as follows: an Independence copula together with a Gaussian copula and either 4 Clayton or 4 Gumbel copulas in all 4 rotations (0°, 90°, 180°, 270°). We select the combination with the lowest WAIC. After that, we take the remaining Clayton/Gumbel copulas one by one and attempt to switch the copula type (Clayton to Gumbel or vise versa). If this switching decreases the WAIC, we keep a better copula type for that rotation and proceed to the next element.

Here we make the assumption, that because Clayton and Gumbel copulas have most of the probability density concentrated in one corner of the unit square (the heavy tail), we can choose the best model for each of the 4 corners independently. When the best combination of Clayton/Gumbel copulas is selected, we can (optionally) reduce the model.

We have not yet used a Frank copula in a heuristic algorithm. We attempt to substitute the Gaussian copula with a Frank copula (if it is still a part of the reduced mixture, see lines 16-19 in Alg. 2). Sometimes, a Gaussian copula can be mistakenly modeled as a Clayton & Gumbel or two Gumbel copulas. So, as a final step (lines 20-31, Alg. 2), we select all pairwise combinations of the remaining elements, and attempt to substitute each of the pairs with a Gaussian copula, selecting the model with the lowest WAIC. Despite a large number of steps in this algorithm, the selection process takes only up to 25 minutes (in case all elements in all rotations are required).

The procedure was designed after observing the model selection process on a variety of synthetic and real neuronal datasets.

C Effective learning rates for different families

The coefficients in the GPLink functions for different copula families are also a part of model hyper-parameters. The choice of these coefficients affects the gradients of the log probability function. Since GPLink functions are nonlinear, they affect the gradients in various parameter ranges to a different extent. This results in variable convergence rates depending on the true copula parameters.

To address the problem of setting up these hyper-parameters, we have created the tests on synthetic data with different copula parameters. Using these tests, we manually adjusted these hyper-parameters such that the GP parameter inference converged in around 1000-2000 iterations for every copula family and parameter range. We have also multiplied the GP values corresponding to the mixture coefficients by 0.5, to effectively slow down the learning of the mixture coefficients ϕ compared to the copula coefficients θ, which also facilitates the convergence.
