# Supplementary material for: Parametric Copula-GP model for analyzing multidimensional neuronal and behavioral relationships
Source: PLoS Comput Biol. 2022 Jan 28;18(1):e1009799. doi: 10.1371/journal.pcbi.1009799 (PMC8827448; doi:10.1371/journal.pcbi.1009799)
Supplement: S2 Text — Include the description of synthetic data generation and the results of mixture model identifiability tests. (PDF) [file pcbi.1009799.s002.pdf]

S2 Text: Model selection tests for "Parametric Copula-GP model for analyzing multidimensional neuronal and behavioral relationships"

January 13, 2022

Computing infrastructure We developed our framework and ran the majority of our experiments (described both in the paper and Supplemental Material) on an Ubuntu 18.04 LTS machine with 2 x Intel Xeon Gold 6142 CPU @ 2.60GHz and 1x GeForce RTX 2080 + 1 x GeForce RTX 2080 Ti GPUs. For training C-vine models, we used another Scientific Linux 7.6 machine with 1 x Intel Xeon Silver 4114 CPU @ 2.20GHz and 8 x GeForce RTX 2080 Ti GPUs.

Model selection for bivariate copulas

Synthetic data We generate artificial data by sampling from a copula mixture, parametrized in two different ways:

1. mixing concentrations of all copulas were constant and equal to $1/N$ (N = number of copulas), but copula parameters θ were parametrized by the phase-shifted sinus functions:

$$\theta_i = A_i \sin\left(\pi m \frac{i}{N} + 2\pi x\right) + B_i, \qquad x \in [0, 1] \tag{1}$$

   where i is the index of the copula in a mixture, $m = 1$. For Clayton and Gumbel copulas, the absolute value of the sinus was used. The amplitudes A_i were chosen to cover most of the range of parameters, except for extremely low or high θs for which all copula families become indistinguishable (from independence or deterministic dependence, respectively).

2. copula parameters θ were constant, but mixing concentrations ϕ were parametrized by the phase-shifted sinus functions (same as Eq. 1, with $A_i = B_i = 1/N$ and $m = 2$). Such parametrization ensures that the sum of all mixing concentrations remains equal to one ($\sum_{i=1}^{N} \phi = 1$). Yet, each ϕ turns to zero somewhere along this trajectory, allowing us to discriminate the models and infer the correct mixture.

Identifiability tests We tested the ability of the model selection algorithms to select the correct mixture of copula models, the same as the one from which the data was generated. We generated 5000 samples with equally spaced unique inputs on [0,1].

Both model selection algorithms were able to correctly select all of the 1-component and most of the 2-component models on simulated data. For simulated data with larger numbers of components (or 2 very similar components), the WAIC of the selected model was either lower (which is possible given a limited number of samples) or close to the WAIC of the correct parametric model. In other words, the difference between the WAIC of the correct model and of the best selected model never exceeded the $\text{WAIC}_{test\_tol} = 0.05$, which we set up as a criteria for passing the test: $\Delta\text{WAIC} < \text{WAIC}_{test\_tol}$. Since all the tests were passed successfully, we conclude that both algorithms are capable of finding optimal or close-to-optimal solutions for copula mixtures.

A more detailed report on the model identifiability tests Tables A-E below illustrate the search for the best model. The copula model names in these tables are shortened to the first two letters, e.g. Gumbel becomes 'Gu', Frank becomes 'Fr'. The information in these Tables provides some intuition on the model selection process and the range of WAICs for the correct or incorrect models. The final selected models are shown in bold.

Table A demonstrates that both greedy and heuristic algorithms can identify the correct single copula model. Some key intermediate models (M in Alg 1-2 in S1 Text) with their WAICs are listed in the table, along with the total duration of simulations (T, in minutes) on RTX 2080Ti for both algorithms.

Table B shows the identification of the mixtures with 2 components, where the copula parameters θ were constant (independent of x) and mixing concentrations ϕ were parameterized by the phase-shifted sinus functions (Eq. 1). All of these models were correctly identified with both algorithms. The mixtures with 2 components, where the copula parameters θ varied harmonically (as in Eq. 1) but the mixing concentrations ϕ were constant, were harder to identify. Table C shows that a few times, each of the algorithms selected a model that was better than the true model ($\mathrm{WAIC}_{best} - \mathrm{WAIC}_{true} < 0$). The greedy algorithm made one mistake, yet the model it selected was very close to optimal. Such misidentification happens due to the limited number of samples in a given synthetic dataset.

Tables D-E show the model selection for 3 component models. Again, as in Tables B-C, either θ or ϕ was constant. Here, the model selection algorithms could rarely identify the correct model (due to overcompleteness of the mixture models), but always selected the one that was very close to optimal: $\mathrm{WAIC}_{best} - \mathrm{WAIC}_{true} \ll \mathrm{WAIC}_{test\_tol}$.

Note, that WAIC_{test_tol} is different from `waic_tol`. We have set `waic_tol` for comparison against Independent model to such a small value (10x smaller than WAIC_{test_tol}) because we want to avoid making false assumptions about conditional independences in the model. Also note, that the WAIC of the true model depends on the particular synthetic dataset generated in each test. Therefore, the final WAIC in the left and in the right columns of Tables A-E can be slightly different (yet, right within WAIC_{test_tol}).

Table A. The model selection histories for 1-element mixtures

True	Greedy			Heuristic		
Model	Search attempts	WAIC	T	Search attempts	WAIC	T
Ga	Ga	-0.1619	25 m	Ga	-0.1513	3 m
	GaFr	-0.1610		InGaGu^{180}Gu^{270}Gu^{0}-Gu^{90}	-0.1499	
	**Ga**	**-0.1619**		InGaCl^{0}Cl^{90}Cl^{180}Cl^{270}	-0.1498	
				**Ga**	**-0.1513**	
Fr	Fr	-0.1389	57 m	Ga	-0.1400	3 m
	FrCl^{90}	-0.1395		InGaGu^{180}Gu^{270}Gu^{0}-Gu^{90}	-0.1391	
	FrCl^{90}Gu^{270}	-0.1396		InGaCl^{0}Cl^{90}Cl^{180}Cl^{270}	-0.1391	
	FrCl^{90}Gu^{270}Gu^{90}	-0.1396		**Fr**	**-0.1509**	
	**Fr**	**-0.1389**				
Cl^{0}	Cl^{0}	-0.5225	37 m	Ga	-0.3825	5 m
	Cl^{0}Gu^{0}	-0.5226		InGaGu^{180}Gu^{270}Gu^{0}-Gu^{90}	-0.4943	
	Cl^{0}Gu^{0}Cl^{180}	-0.5225		InGaCl^{0}Cl^{90}Cl^{180}Cl^{270}	-0.5303	
	**Cl^{0}**	**-0.5224**		**Cl^{0}**	**-0.5311**	
Gu^{0}	Gu^{0}	-0.6267	43 m	Ga	-0.5555	7 m
	Gu^{0}Cl^{180}	-0.6268		InGaGu^{180}Gu^{270}Gu^{0}-Gu^{90}	-0.5988	
	Gu^{0}Cl^{180}Gu^{180}	-0.6267		InGaCl^{0}Cl^{90}Cl^{180}Cl^{270}	-0.5946	
	**Gu^{0}**	**-0.6230**		GaGu^{0}	-0.6040	
				**Gu^{0}**	**-0.6050**	
Cl^{90}	Cl^{90}	-0.5389	22 m	Ga	-0.3922	5 m
	Cl^{90}Cl^{270}	-0.5389		InGaGu^{180}Gu^{270}Gu^{0}-Gu^{90}	-0.5047	
	**Cl^{90}**	**-0.5389**		InGaCl^{0}Cl^{90}Cl^{180}Cl^{270}	-0.5409	
				**Cl^{90}**	**-0.5410**	
Gu^{90}	Gu^{90}	-0.6137	55 m	Ga	-0.5501	7 m
	Gu^{90}Gu^{270}	-0.6144		InGaGu^{180}Gu^{270}Gu^{0}-Gu^{90}	-0.5893	
	Gu^{90}Gu^{270}Cl^{270}	-0.6145		InGaCl^{0}Cl^{90}Cl^{180}Cl^{270}	-0.5831	
	Gu^{90}Gu^{270}Cl^{270}Cl^{90}	-0.6144		GaGu^{90}	-0.5887	
	**Gu^{90}**	**-0.6137**		**Gu^{90}**	**-0.5950**	
Cl^{180}	Cl^{180}	-0.5566	36 m	Ga	-0.3932	7 m
	Cl^{180}Cl^{0}	-0.5582		InGaGu^{180}Gu^{270}Gu^{0}-Gu^{90}	-0.4956	
	Cl^{180}Cl^{0}In	-0.5582		InGaCl^{0}Cl^{90}Cl^{180}Cl^{270}	-0.5493	
	**Cl^{180}**	**-0.5565**		**Cl^{180}**	**-0.5489**	
Gu^{180}	Gu^{180}	-0.6131	43 m	Ga	-0.5553	6 m
	Gu^{180}Cl^{0}	-0.6164		InGaGu^{180}Gu^{270}Gu^{0}-Gu^{90}	-0.6091	
	Gu^{180}Cl^{0}Fr	-0.6163		InGaCl^{0}Cl^{90}Cl^{180}Cl^{270}	-0.6045	
	**Gu^{180}**	**-0.6131**		**Gu^{180}**	**-0.6154**	
Cl^{270}	Cl^{270}	-0.5434	23 m	Ga	-0.3909	5 m
	Cl^{270}Gu^{270}	-0.5433		InGaGu^{180}Gu^{270}Gu^{0}-Gu^{90}	-0.5094	
	**Cl^{270}**	**-0.5434**		InGaCl^{0}Cl^{90}Cl^{180}Cl^{270}	-0.5535	
				**Cl^{270}**	**-0.5548**	

True Model	Greedy			Heuristic		
	Search attempts	WAIC	T	Search attempts	WAIC	T
Gu^{270}	Gu^{270}	-0.5928	51 m	Ga	-0.5763	6 m
	$Gu^{270}Cl^{90}$	-0.5934		$InGaGu^{180}Gu^{270}Gu^{0}Gu^{90}$	-0.6277	
	$Gu^{270}Cl^{90}In$	-0.5935		$InGaCl^{0}Cl^{90}Cl^{180}Cl^{270}$	-0.6179	
	$Gu^{270}Cl^{90}InCl^{180}$	-0.5931		**Gu^{270}**	**-0.6300**	
	**Gu^{270}**	**-0.5928**				

Table B. The model selection histories for 2-element mixtures with constant θ and variable ϕ

True Model	Greedy			Heuristic		
	Search attempts	WAIC	T	Search attempts	WAIC	T
Gu^{90} Ga	Ga	-0.1877	101 m	Ga	-0.1922	11 m
	$GaGu^{90}$	-0.2855		$InGaGu^{180}Gu^{270}Gu^{0}Gu^{90}$	-0.3070	
	$GaGu^{90}Cl^{270}$	-0.2855		$InGaCl^{0}Cl^{90}Cl^{180}Cl^{270}$	-0.2996	
	$GaGu^{90}Cl^{270}Fr$	-0.2856		$GaCl^{0}Gu^{0}Gu^{90}$	-0.3082	
	$GaGu^{90}Cl^{270}FrGu^{270}$	-0.2856		$GaCl^{0}Cl^{180}Gu^{90}$	-0.3076	
	$GaGu^{90}Cl^{270}FrGu^{270}Cl^{90}$	-0.2856		**$GaGu^{90}$**	**-0.3091**	
	**$Gu^{90}Ga$**	**-0.2854**				
Ga Cl^{270}	Fr	-0.1635	87 m	Ga	-0.1600	5 m
	$FrCl^{270}$	-0.2707		$InGaGu^{180}Gu^{270}Gu^{0}Gu^{90}$	-0.2687	
	$FrCl^{270}Ga$	-0.2747		$InGaCl^{0}Cl^{90}Cl^{180}Cl^{270}$	-0.2835	
	$FrCl^{270}GaGu^{180}$	-0.2782		**$GaCl^{270}$**	**-0.2845**	
	$FrCl^{270}GaGu^{180}Cl^{90}$	-0.2781				
	**$GaCl^{270}$**	**-0.2821**				
Gu^{180} Fr	Gu^{180}	-0.1681	99 m	Ga	-0.1534	8 m
	$Gu^{180}Fr$	-0.2099		$InGaGu^{180}Gu^{270}Gu^{0}Gu^{90}$	-0.1993	
	$Gu^{180}FrCl^{180}$	-0.2101		$InGaCl^{0}Cl^{90}Cl^{180}Cl^{270}$	-0.1977	
	$Gu^{180}FrCl^{180}Cl^{90}$	-0.2105		$InGaGu^{180}$	-0.2074	
	$Gu^{180}FrCl^{180}Cl^{90}In$	-0.2106		**$FrGu^{180}$**	**-0.2104**	
	$Gu^{180}FrCl^{180}Cl^{90}InGu^{270}$	-0.2099				
	**$FrGu^{180}$**	**-0.2099**				
Cl^{0} Cl^{90}	Fr	-0.1587	92 m	Ga	-0.1652	5 m
	$FrCl^{0}$	-0.2600		$InGaGu^{180}Gu^{270}Gu^{0}Gu^{90}$	-0.3142	
	$FrCl^{0}Cl^{90}$	-0.3173		$InGaCl^{0}Cl^{90}Cl^{180}Cl^{270}$	-0.3430	
	$FrCl^{0}Cl^{90}Gu^{270}$	-0.3176		**$Cl^{0}Cl^{90}$**	**-0.3448**	
	$FrCl^{0}Cl^{90}Gu^{270}In$	-0.3176				
	$FrCl^{0}Cl^{90}Gu^{270}InCl^{270}$	-0.3175				
	**$Cl^{90}Cl^{0}$**	**-0.3190**				
Cl^{180} Gu^{270}	Fr	-0.2204	103 m	Ga	-0.1965	7 m
	$FrCl^{180}$	-0.3488		$InGaGu^{180}Gu^{270}Gu^{0}Gu^{90}$	-0.3591	
	$FrCl^{180}Gu^{270}$	-0.3874		$InGaCl^{0}Cl^{90}Cl^{180}Cl^{270}$	-0.3688	
	$FrCl^{180}Gu^{270}Cl^{90}$	-0.3877		$GaGu^{270}Cl^{180}$	-0.3771	
	$FrCl^{180}Gu^{270}Cl^{90}Ga$	-0.3878		**$Gu^{270}Cl^{180}$**	**-0.3772**	
	$FrCl^{180}Gu^{270}Cl^{90}GaGu^{90}$	-0.3878				
	**$Gu^{270}Cl^{180}$**	**-0.3888**				

Table C. The model selection histories for 2-element mixtures with constant ϕ and variable θ

True	Greedy			Heuristic		
Model	Search attempts	WAIC	T	Search attempts	WAIC	T
Gu^{90}	Gu^{90}	-0.1419	60 m	Ga	-0.1538	10 m
Ga	Gu^{90}Fr	-0.2022		InGaGu^{180}Gu^{270}Gu^{0}-Gu^{90}	-0.2320	
	Gu^{90}FrCl^{270}	-0.2024		InGaCl^{0}Cl^{90}Cl^{180}Cl^{270}	-0.2218	
	Gu^{90}FrCl^{270}Ga	-0.2024		GaCl^{90}Gu^{0}Gu^{90}	-0.2321	
	**FrGu^{90}**	**-0.2021**		**GaGu^{90}**	**-0.2326**	
	$\mathrm{WAIC}_{best} - \mathrm{WAIC}_{true}$:	**-0.0013**				
Ga	Gu^{90}	-0.1495	56 m	Ga	-0.1062	7 m
Cl^{270}	Gu^{90}Fr	-0.1894		InGaGu^{180}Gu^{270}Gu^{0}-Gu^{90}	-0.1747	
	Gu^{90}FrCl^{270}	-0.1915		InGaCl^{0}Cl^{90}Cl^{180}Cl^{270}	-0.1783	
	Gu^{90}FrCl^{270}In	-0.1902		GaGu^{0}Cl^{270}	-0.1812	
	**Cl^{270}FrGu^{90}**	**-0.1915**		**GaCl^{270}**	**-0.1801**	
	$\mathrm{WAIC}_{best} - \mathrm{WAIC}_{true}$:	**0.0032**				
Gu^{180}	Gu^{180}	-0.1600	58 m	Ga	-0.1331	8 m
Fr	Gu^{180}Fr	-0.2191		InGaGu^{180}Gu^{270}Gu^{0}-Gu^{90}	-0.1944	
	Gu^{180}FrCl^{270}	-0.2195		InGaCl^{0}Cl^{90}Cl^{180}Cl^{270}	-0.1936	
	Gu^{180}FrCl^{270}Cl^{0}	-0.2190		GaGu^{180}Cl^{90}Gu^{0}Gu^{90}	-0.1945	
	**FrGu^{180}**	**-0.2190**		**GaGu^{180}**	**-0.1992**	
				$\mathrm{WAIC}_{best} - \mathrm{WAIC}_{true}$:	**-0.0094**	
Cl^{0}	Gu^{180}	-0.0253	62 m	Ga	-0.0079	5 m
Cl^{90}	Gu^{180}Cl^{90}	-0.2383		InGaGu^{180}Gu^{270}Gu^{0}-Gu^{90}	-0.1904	
	Gu^{180}Cl^{90}Cl^{0}	-0.2506		InGaCl^{0}Cl^{90}Cl^{180}Cl^{270}	-0.2330	
	Gu^{180}Cl^{90}Cl^{0}In	-0.2509		**Cl^{0}Cl^{90}**	**-0.2361**	
	Gu^{180}Cl^{90}Cl^{0}InFr	-0.2508				
	**Cl^{0}Cl^{90}**	**-0.2586**				
Cl^{180}	Gu^{270}	-0.0242	69 m	Ga	-0.0083	6 m
Gu^{270}	Gu^{270}Cl^{180}	-0.2499		InGaGu^{180}Gu^{270}Gu^{0}-Gu^{90}	-0.2277	
	Gu^{270}Cl^{180}Gu^{180}	-0.2517		InGaCl^{0}Cl^{90}Cl^{180}Cl^{270}	-0.2535	
	Gu^{270}Cl^{180}Gu^{180}In	-0.2518		**GaCl^{90}Cl^{180}**	**-0.2549**	
	Gu^{270}Cl^{180}Gu^{180}InCl^{0}	-0.2518				
	Gu^{270}Cl^{180}Gu^{180}InCl^{0}-Fr	-0.2518				
	**Cl^{180}Gu^{270}**	**-0.2500**				
				$\mathrm{WAIC}_{best} - \mathrm{WAIC}_{true}$:	**-0.0098**	

Table D. The model selection histories for 3-element mixtures with constant θ and variable ϕ

True	Greedy			Heuristic		
Model	Search attempts	WAIC	T	Search attempts	WAIC	T
Ga	Gu^{0}	-0.1399	44 m	Ga	-0.1252	6 m
Cl^{90}	$Gu^{0}Cl^{90}$	-0.2494		$InGaGu^{180}Gu^{270}Gu^{0}$-$Gu^{90}$	-0.2481	
Gu^{0}	$Gu^{0}Cl^{90}Cl^{0}$	-0.2519		$InGaCl^{0}Cl^{90}Cl^{180}Cl^{270}$	-0.2565	
	$Gu^{0}Cl^{90}Cl^{0}Fr$	-0.2518		**$GaCl^{90}Cl^{180}$**	**-0.2564**	
	**$Cl^{90}Gu^{0}$**	**-0.2494**				
	$WAIC_{best} - WAIC_{true}$:	**-0.0036**		$WAIC_{best} - WAIC_{true}$:	**0.0014**	
Fr	Fr	-0.0591	77 m	Ga	-0.0489	6 m
Cl^{90}	$FrCl^{90}$	-0.1460		$InGaGu^{180}Gu^{270}Gu^{0}$-$Gu^{90}$	-0.1573	
Gu^{0}	$FrCl^{90}Gu^{0}$	-0.1730		$InGaCl^{0}Cl^{90}Cl^{180}Cl^{270}$	-0.1578	
	$FrCl^{90}Gu^{0}Cl^{180}$	-0.1736		**$GaCl^{90}Cl^{180}$**	**-0.1621**	
	$FrCl^{90}Gu^{0}Cl^{180}In$	-0.1734				
	**$Gu^{0}Cl^{90}Fr$**	**-0.1731**				
				$WAIC_{best} - WAIC_{true}$:	**0.0059**	
Fr	Fr	-0.0741	87 m	Ga	-0.0618	9 m
Cl^{180}	$FrCl^{180}$	-0.1513		$InGaGu^{180}Gu^{270}Gu^{0}$-$Gu^{90}$	-0.1567	
Gu^{270}	$FrCl^{180}Gu^{270}$	-0.1707		$InGaCl^{0}Cl^{90}Cl^{180}Cl^{270}$	-0.1670	
	$FrCl^{180}Gu^{270}Cl^{90}$	-0.1708		$InGaGu^{270}Cl^{180}Cl^{270}$	-0.1680	
	$FrCl^{180}Gu^{270}Cl^{90}$-$Gu^{180}$	-0.1711		$InGaGu^{270}Cl^{180}Gu^{90}$	-0.1695	
	$FrCl^{180}Gu^{270}Cl^{90}$-$Gu^{180}Cl^{0}$	-0.1710		**$InGu^{270}Cl^{180}$**	**-0.1735**	
	**$Gu^{270}Cl^{180}Fr$**	**-0.1703**				
				$WAIC_{best} - WAIC_{true}$:	**-0.0011**	
Gu^{0}	Gu^{0}	-0.1695	47 m	Ga	-0.1477	11 m
Gu^{180}	$Gu^{0}Cl^{90}$	-0.3040		$InGaGu^{180}Gu^{270}Gu^{0}$-$Gu^{90}$	-0.2986	
Cl^{90}	$Gu^{0}Cl^{90}Gu^{180}$	-0.3234		$InGaCl^{0}Cl^{90}Cl^{180}Cl^{270}$	-0.3033	
	$Gu^{0}Cl^{90}Gu^{180}Cl^{180}$	-0.3233		$GaGu^{180}Cl^{90}Cl^{180}$	-0.3054	
	**$Gu^{180}Cl^{90}Gu^{0}$**	**-0.3234**		$GaGu^{180}Cl^{90}Gu^{0}$	-0.3111	
				**$Gu^{180}Cl^{90}Gu^{0}$**	**-0.3113**	

Table E. The model selection histories for 3-element mixtures with constant ϕ and variable θ

True Model	Greedy Search attempts	Greedy WAIC	Greedy T	Heuristic Search attempts	Heuristic WAIC	Heuristic T
Ga	Fr	-0.0177	66 m	Ga	-0.0142	13 m
Cl^{90}	$FrGu^{270}$	-0.1284		$InGaGu^{180}Gu^{270}Gu^{0}$-$Gu^{90}$	-0.1291	
Gu^{0}	$FrGu^{270}Gu^{0}$	-0.1407		$InGaCl^{0}Cl^{90}Cl^{180}Cl^{270}$	-0.1289	
	$FrGu^{270}Gu^{0}Cl^{0}$	-0.1423		$InCl^{0}Gu^{270}Gu^{0}$	-0.1317	
	$FrGu^{270}Gu^{0}Cl^{0}Cl^{180}$	-0.1435		$InCl^{0}Cl^{90}Gu^{0}$	-0.1346	
	$FrGu^{270}Gu^{0}Cl^{0}Cl^{180}In$	-0.1432		$InCl^{0}Cl^{90}Cl^{180}$	-0.1301	
	**$Gu^{0}Gu^{270}$**	**-0.1451**		**$GaCl^{90}Cl^{180}$**	**-0.1313**	
	$WAIC_{best} - WAIC_{true}$:	**0.0132**		$WAIC_{best} - WAIC_{true}$:	**0.0068**	
Fr	Fr	-0.0265	71 m	Ga	-0.0192	9 m
Cl^{90}	$FrGu^{270}$	-0.1290		$InGaGu^{180}Gu^{270}Gu^{0}$-$Gu^{90}$	-0.1411	
Gu^{0}	$FrGu^{270}Gu^{0}$	-0.1445		$InGaCl^{0}Cl^{90}Cl^{180}Cl^{270}$	-0.1429	
	$FrGu^{270}Gu^{0}Cl^{180}$	-0.1450		$InGaGu^{180}Cl^{90}Cl^{180}$	-0.1474	
	$FrGu^{270}Gu^{0}Cl^{180}Cl^{0}$	-0.1466		$InGaGu^{180}Cl^{90}Gu^{0}$	-0.1472	
	$FrGu^{270}Gu^{0}Cl^{180}Cl^{0}In$	-0.1468		**$InCl^{90}Gu^{0}$**	**-0.1477**	
	**$Gu^{0}Gu^{270}$**	**-0.1451**				
	$WAIC_{best} - WAIC_{true}$:	**0.0109**		$WAIC_{best} - WAIC_{true}$:	**0.0010**	
Fr	Fr	-0.0129	61 m	Ga	-0.0185	6 m
Cl^{180}	$FrGu^{270}$	-0.1105		$InGaGu^{180}Gu^{270}Gu^{0}$-$Gu^{90}$	-0.1309	
Gu^{270}	$FrGu^{270}Gu^{0}$	-0.1237		$InGaCl^{0}Cl^{90}Cl^{180}Cl^{270}$	-0.1326	
	$FrGu^{270}Gu^{0}Cl^{180}$	-0.1254		$InGu^{270}Cl^{180}$	-0.1393	
	$FrGu^{270}Gu^{0}Cl^{180}$-Gu^{180}	-0.1248		$InGu^{270}Gu^{0}$	-0.1334	
	**$Gu^{0}Gu^{270}Fr$**	**-0.1234**		**$InGu^{270}Gu^{0}$**	**-0.1326**	
	$WAIC_{best} - WAIC_{true}$:	**0.0094**		$WAIC_{best} - WAIC_{true}$:	**0.0088**	
Gu^{0}	Gu^{0}	-0.0756	55 m	Ga	-0.0454	7 m
Gu^{180}	$Gu^{0}Cl^{90}$	-0.2380		$InGaGu^{180}Gu^{270}Gu^{0}$-$Gu^{90}$	-0.2476	
Cl^{90}	$Gu^{0}Cl^{90}Cl^{0}$	-0.2556		$InGaCl^{0}Cl^{90}Cl^{180}Cl^{270}$	-0.2459	
	$Gu^{0}Cl^{90}Cl^{0}Ga$	-0.2591		$GaCl^{0}Gu^{270}Gu^{0}$	-0.2493	
	$Gu^{0}Cl^{90}Cl^{0}GaCl^{270}$	-0.2590		$GaCl^{0}Cl^{90}Gu^{0}$	-0.2559	
	**$Cl^{0}Cl^{90}Gu^{0}$**	**-0.2555**		**$Cl^{0}Cl^{90}Gu^{0}$**	**-0.2538**	
	$WAIC_{best} - WAIC_{true}$:	**0.0026**		$WAIC_{best} - WAIC_{true}$:	**0.0006**	
