# Supplementary material for: Parametric Copula-GP model for analyzing multidimensional neuronal and behavioral relationships
Source: PLoS Comput Biol. 2022 Jan 28;18(1):e1009799. doi: 10.1371/journal.pcbi.1009799 (PMC8827448; doi:10.1371/journal.pcbi.1009799)
Supplement: S3 Text — Describes validation of entropy estimation for Gaussian and Clayton copulas, validation on UCI benchmarks, comparison between single copula and mixture models, and additional tests that explore limitations of empirical marginal estimation. (PDF) [file pcbi.1009799.s003.pdf]

S3 Text: Supplementary validation for "Parametric Copula-GP model for analyzing multidimensional neuronal and behavioral relationships"

January 10, 2022

Contents

A Accuracy of entropy estimation **1**

B Validation on a non-gaussian copula **2**

C Performance on a UCI benchmark dataset **2**

D Limitations of empirical marginal estimation **3**

D.1 Robustness of non-parametric estimation of marginal distributions . . . 3

D.2 Sensitivity of copula parameter estimation to errors in eCDF estimation 4

A Accuracy of entropy estimation

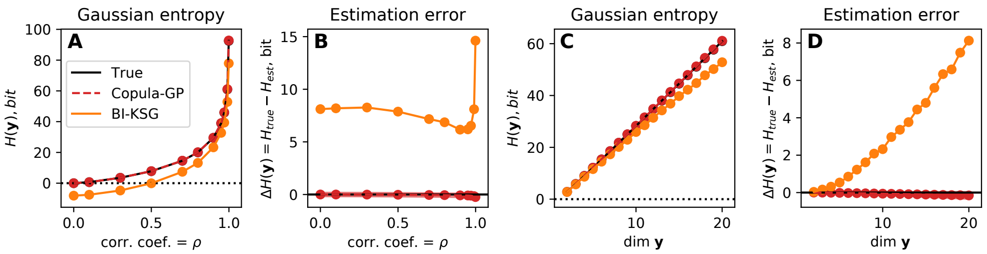

Fig A. Accuracy of the entropy estimation for multivariate Gaussian distributions. **A** Entropy of the 20-dimensional multivariate Gaussian distribution for different correlation coefficients ρ. **B** Estimation error for the entropy shown in A. **C** Entropy of the multivariate Gaussian distributions with $\rho = 0.99$ and varying dimensionality. **D** Estimation error for the entropy shown in C.

In this section, we consider a fixed copula mixture model with known parameters θ and test the reliability of the entropy estimation with Monte Carlo (MC) integration. We test the accuracy of the entropy estimation on a multivariate Gaussian distribution, with $\mathrm{cov}(y_i, y_j) = \rho + (1-\rho)\,\delta_{ij}$, where δ_{ij} is Kronecker's delta and $\rho \in [0, 0.999]$. Given a known Gaussian copula, we estimate the entropy with MC integration and

compare it to the analytically calculated true value. We set up a tolerance to $\Delta H = 0.01(\dim \mathbf{y})$. As a result, for every correlation ρ (Fig AA-B) and every number of dimensions $\dim \mathbf{y}$ (Fig AC-D), the Copula-GP provides an accurate result, within the error margin. In Figure A, BI-KSG estimates [1] obtained on the dataset with 10k samples are shown for comparison. This experiment 1) validates the MC integration; 2) validates the numerical stability of the probability density function of the Gaussian copula up to a specified maximal $\rho = 0.999$ (for $\rho > 0.999$ the model is indistinguishable from the deterministic dependence $u_1 = u_2$).

B Validation on a non-gaussian copula

Copula-GP is guaranteed to produce unbiased entropy estimates when the true dependency matches the parametric model (i.e. is Gaussian, Clayton, Frank or Gumbel, or any linear mixture of those), assuming that the implementation of log-likelihood and sampling is correct, which is ensured by the tests and correctly estimated parameters.

Fig 3A tested the entropy and mutual information estimates on the Gaussian copula. Here, we follow the same procedure and generate samples from a C-Vine, in which each bivariate element is a Clayton copula. We varied the parameter θ of the Clayton copula linearly from 0 to 2.

The observed result is qualitatively identical to the validation on a Gaussian copula model (see Fig 3A): Copula-GP measures both entropy and mutual information exactly (within integration tolerance, see Fig B), while the performance of the non-parametric methods on this dataset is lower.

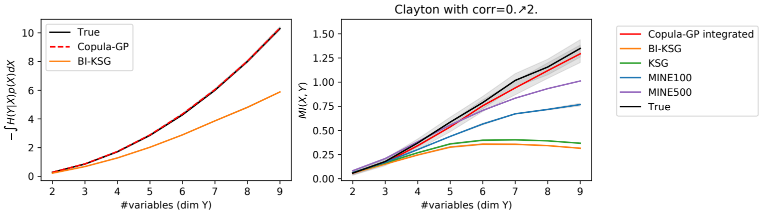

Fig B. Conditional entropy $H(\mathbf{y}|x)$ (left panel) and mutual information $I(x, \mathbf{y})$ (right panel) measured by different methods on synthetic data, generated from Clayton copula. The performance of various estimators is qualitatively similar to Fig 3A with the Gaussian copula.

C Performance on a UCI benchmark dataset

Here we compare our method with the similar method from Lopez-Paz et al. [2] and Hernández-Lobato et al. [3]. Apart from differences in implementation (SVI vs. EP, (un)/conditional marginals), our Copula-GP method has two conceptual improvements 1) we use non-parametric **conditional** marginals, instead of unconditional; 2) we use linear mixtures of copula elements with qualitatively different tail dependencies. Using the UCI *shuttle* dataset [4], we compare the performance of the Lopez-Paz model vs. Copula-GP model which was either allowed to use only one copula element, or a mixture of any number of elements (selected with the model selection method, see S1 Methods). We used vine copulas truncated at 3 or 5 trees, ran them on the training set and evaluated the log-likelihood on the test set (higher is better).

As a result, Copula-GP restricted to the use of only one copula element produced similar results to Lopez-Paz et al. [2], while our mixture model considerably outperformed both of these single element models.

Table A1. Average test log-likelihood for Copula-GP vs. Lopez-Paz et al. [2]

Trees	Lopez-Paz et al.	Single copula	Mixture
3	3.645±0.427	3.759±0.307	**4.326±0.170**
9	4.755±0.389	4.470±0.215	**5.115±0.150**

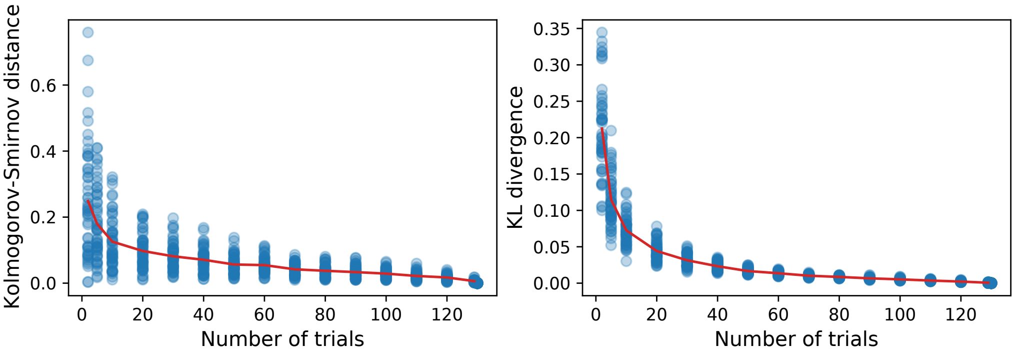

Fig C. The discrepancy between the estimated density $p_N(y_i|x)$ (based on N trials) and the most accurate estimate $p_{all}(y_i|x)$ (based on all 127 trials). Left plot: Kolmogorov-Smirnov distance; right plot: Kullback-Leibler divergence.

D Limitations of empirical marginal estimation

In this section, we explore the limitations of the empirical non-parametric estimation of marginal distributions (eCDF). First, we estimate the errors in those estimations given the limited amount of data (i.e. few trials). Second, we show how these errors in eCDFs propagate through the model and affect the copula parameter estimation. In conclusion, we show that the number of trials in our VR experiments was sufficient for empirical CDF estimation.

D.1 Robustness of non-parametric estimation of marginal distributions

The number of samples available for estimating the statistics of neural responses given a certain phase of the experiment $p(\mathbf{y}|x)$ is equal to the number of experimental trials, which is often quite limited. In the experimental data that we were working with, there were 127 trials in total. Here we are testing whether this amount of data is sufficient for estimating empirical marginal distributions $p(y_i|x)$. Note, that we still assume that this number of samples is insufficient for estimating *joint* distributions of neural responses conditioned on time $p(\mathbf{y}|x)$ without further assumptions. These assumptions are formalized in parametric copula models (Fig 1 in main text).

To show that the number of trials in the experimental dataset is sufficient for estimating the conditional marginals, we performed the following analysis. We first estimated the conditional probability density function $p_{all}(y_i|x)$ for one neuron (e.g. neuron $i = 3$, see Fig 5B) using the fastKDE algorithm and the data from all trials. Next, we consider different subsets of N trials, taken at random (without replacement, 50 independent subsamples), and estimated the conditional pdf $p_N(y_i|x)$ again. For each estimate, we compute the distance from an estimated density $p_N(y_i|x)$ to the most accurate estimate $p_{all}(y_i|x)$, using Kolmogorov-Smirnov distance and KL divergence (Fig C).

Both measures of the difference between estimated densities decay with the increase of the number of trials and eventually saturate. The Kolmogorov-Smirnov distance is a supremum on a difference between CDFs. Since we use the conditional CDF values for

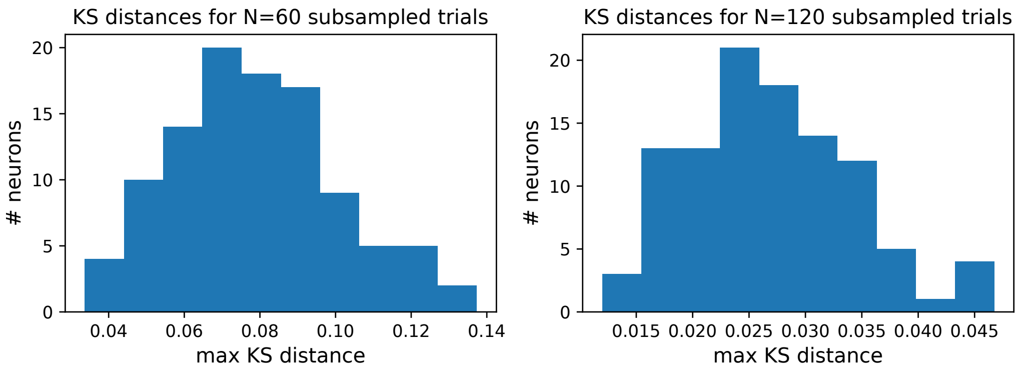

Fig D. Distributions of the maximal Kolmogorov-Smirnov (KS) distances across all neurons for $n = 60$ and $n = 120$ subsampled trials.

mapping the data on the unit square, the KS distance corresponds to the error in the coordinates of the datapoints in the copula space. If our estimate is inaccurate, the mapping will be incorrect, which will add noise to the datapoints $u^x = CDF(y_i|x)$, which we then model with copulas. One can see from the plot above that, on average across shuffles, the maximal error in a CDF drops below 0.1 already for 20 trials (for neuron 3). For subsets with more than 80 trials there were only a few shuffles that had a maximal error greater than 0.1.

We time fixed the number of subsampled trials and repeated the analysis for all of the neurons in the dataset (Fig D). For the majority of neurons, the maximal KS distance (i.e. the KS distance for the worst-case subsample) was around 0.08 for n=60 and 0.03 for $n = 120$ (n – subsampled trials), and never exceeded 0.14 for $n = 60$ and 0.05 for $n = 120$ (Fig D). From this analysis, we conclude that the estimation of conditional CDFs is robust and the errors are small (< 0.1 in copula space) for the number of trials we have available in the dataset (127).

In applications to other datasets where the number of trials is much smaller (e.g. 10), we suggest finding a suitable class of parametric marginal distributions. However, distinguishing between different shapes of dependence (e.g. Gaussian vs. Clayton) could also become difficult with such scarce data. Therefore, our model is best suited for relatively large datasets (~ 100 trials or more).

D.2 Sensitivity of copula parameter estimation to errors in eCDF estimation

In this section, we study the sensitivity of the estimated copula parameters to the noise that comes from erroneous estimates of the marginals. To answer this question systematically, we have perturbed every CDF estimate at a random location x_p, shifting the CDF value up or down by a fixed step ε. The rest of the function: $\mathrm{CDF}(x; x > x_p)$ for 'up' perturbation or $\mathrm{CDF}(x; x < x_p)$ for down perturbation, was scaled linearly such that the CDF values remain within the range $[0, 1]$. We have then varied the magnitude of a fixed step ε and measured the error in the copula parameter estimate.

For this analysis, we used the synthetic data from Figure 3, which is well described by a single copula. We have observed that for $\varepsilon <= 0.05$ (e.g. $\varepsilon = 0.05$ or $\varepsilon = 0.02$ in Fig E), most of the random perturbations do not affect the estimate of the copula parameter. This magnitude of the perturbation (ε) can be related back to the Kolmogorov-Smirnov distance between estimated CDF based on subsampled trials (see previous section). We conclude that given the amount of data we have (127 trials), the error in CDF estimates is well below 0.05 level.

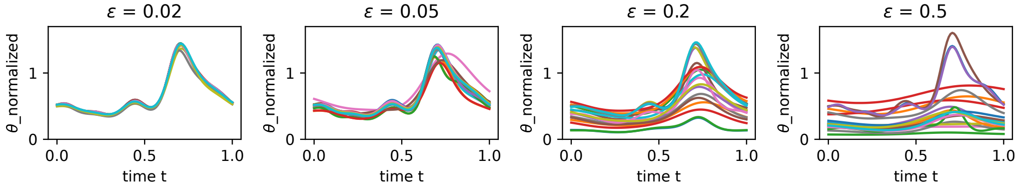

Fig E. Sensitivity of GP estimates to eCDF errors. The plots show samples from a Gaussian Process fitted to the data that was transformed using perturbed empirical CDFs. The title of each plot indicates the magnitude of perturbation ε, which corresponds to the KS distance estimated in a previous section.

References

1. Gao W, Oh S, Viswanath P. Demystifying Fixed k-Nearest Neighbor Information Estimators; 2016.

2. Lopez-Paz D, Hernández-Lobato JM, Zoubin G. Gaussian process vine copulas for multivariate dependence. In: International Conference on Machine Learning; 2013. p. 10–18.

3. Hernández-Lobato JM, Lloyd JR, Hernández-Lobato D. Gaussian Process Conditional Copulas with Applications to Financial Time Series. In: Burges CJC, Bottou L, Welling M, Ghahramani Z, Weinberger KQ, editors. Advances in Neural Information Processing Systems 26. Curran Associates, Inc.; 2013. p. 1736–1744. Available from: `http://papers.nips.cc/paper/5084-gaussian-process-conditional-copulas-with-applications-to-financial-time-serie` `pdf`.

4. Frank A, Asuncion A, et al. UCI machine learning repository, 2010. URL http://archive ics uci edu/ml. 2011;15:22.
