# Supplementary material for: Parametric Copula-GP model for analyzing multidimensional neuronal and behavioral relationships
Source: PLoS Comput Biol. 2022 Jan 28;18(1):e1009799. doi: 10.1371/journal.pcbi.1009799 (PMC8827448; doi:10.1371/journal.pcbi.1009799)
Supplement: S4 Text — Includes additional parameter visializations and an ablation study for the copulas in the mixtures shown in Fig 5. (PDF) [file pcbi.1009799.s004.pdf]

S4 Text: Interpretation of Copula-GP models for “Parametric Copula-GP model for analyzing multidimensional neuronal and behavioral relationships”

January 10, 2022

Contents

A Model parameters for the bivariate neuronal and behavioral examples 1

B Finding important dependencies: ablation study 2

C Finding important data points: heavy tail detection 3

A Model parameters for the bivariate neuronal and behavioral examples

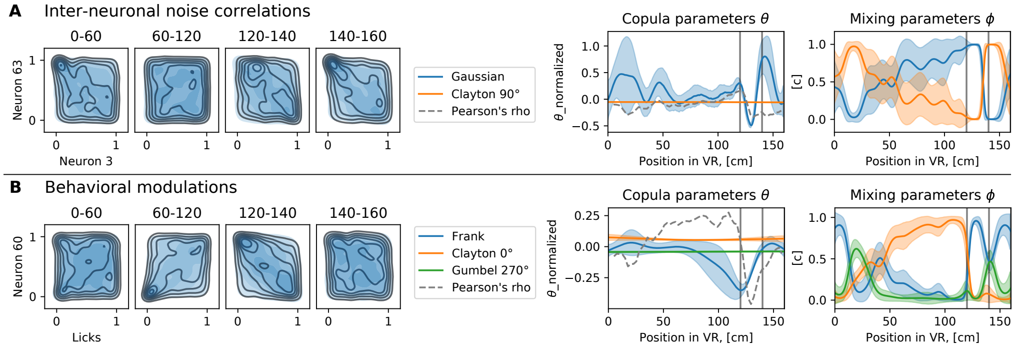

Fig A. Parameters of the copula mixture models. From left to right: copula probability densities (same as Fig 4C-D); a list of selected copula elements; copula parameters θ; mixing concentrations ϕ. These plots are provided for: **i.** the noise correlation example; **ii.** the behavioral modulation example.

In this section, we provide visualisations for the parameters of the bivariate copula models from Fig 4C-F and discuss the interpretability of these models.

Fig A shows the probability density of the joint distribution of two variables and the parameters of a corresponding Copula-GP mixture model. The plots on the left repeat

Fig 4C-D and represent the true density (outlines) and the copula model density (blue shades) for each part of the task.

In the noise correlation example (Fig Ai), we observe the *tail dependencies* between the variables (i.e. concentration of the probability density in a corner of the unit square) around [0-60] cm and [140-160] cm of the virtual corridor. There is only one element with a tail dependency in this mixture: Clayton 90° copula. On the right-most plot in Fig Ai, we see the mixing concentration for the elements of the mixture model. The concentration of Clayton 90° copula (orange line) is close to 100% around 20 cm and 150 cm, which agrees with our observations from the density plots.

The confidence intervals ($\pm 2\sigma$) for the parameters approximated with Gaussian processes are shown with shaded areas in parameter plots. These intervals provide a measure of uncertainty in model parameters. For instance, when the concentration of the Gaussian copula in the mixture is close to 0% (x around 20 cm and 150 cm), the confidence intervals for the Gaussian copula parameter (θ, blue shade) in Fig Ai become very wide (from almost 0 to 1). Since this copula element is not affecting the mixture for those values of x, its θ parameter has no effect on the mixture model log probability. Therefore, this parameter is not constrained to any certain value. In a similar manner, we see that the variables are almost independent between 60 and 120 cm (see density plots on the left in Fig A). Both copula elements can describe this independence. As a result, the mixing concentrations for both elements have high uncertainty in that interval of x. Yet, Gaussian copula with a slightly positive correlation is still a bit more likely to describe the data in that interval.

The copula parameter plot in Fig Ai also shows Pearson's ρ, which does not change much in this example and remains close to zero. This illustrates, that the traditional linear noise correlation analysis would ignore (or downplay) this pair of neurons as the ones with no dependence. This happens because the Pearson's ρ only captures the linear correlation and ignores the tail dependencies, whereas our model provides a more detailed description of the joint bivariate distribution.

In the behavioral modulation example (Fig Aii), we observe more complicated tail dependencies in the density plots. The best selected model supports this observation and provides a mixture model with 3 components, 2 of which have various tail dependencies. The Clayton 0° copula (orange) describes the lower tail dependence observed in the second part of the virtual corridor with gratings (around [60-120] cm, see Fig 4A for task structure). This dependence can be verbally interpreted as follows: *when there is* ***no*** *licking, the Neuron 60 is certainly silent; but when the animal* ***is*** *licking, the activity of Neuron 60 is slightly positively correlated with the licking rate.*

These examples illustrate, that by analysing the copula parameters and the mixing concentrations of the Copula-GP mixture model, one can interpret the changes in the bivariate dependence structure. Just like traditional *tuning curves* characterize the response of a single neuron, our mixture model characterizes the 'tuning' of the dependence structure between pairs of variables to a given stimulus or context. Knowing the qualitative properties of the copula elements that constitute a copula mixture, one can focus on the dominant element of the copula mixture for every given conditioning variable x and describe the shape of the dependence.

B Finding important dependencies: ablation study

After the model is selected, one can ablate the elements one by one and check the WAIC of these models. Following this procedure, one can test whether all of the elements are important in the mixture.

We performed the ablation on the models from Fig A. All models after ablation had higher WAIC than the originally selected model. Ablation of the Clayton copula in

Fig Aii increased WAIC the most (from -0.043 to -0.033), meaning that the heavy-tailed dependence, modelled by Clayton copula, is essential for describing the relationship between these two neurons. In Fig Ai, both elements were equally important (WAIC after ablation was the same (within tolerance)).

C Finding important data points: heavy tail detection

Since the Copula-GP framework is based on a linear mixture model, it is possible to calculate the probability that a certain data-point was generated from a heavy-tailed component of the distribution (e.g. Clayton). Given a mixture model with estimated parameters, described by:

$$c\left(\mathbf{u}|x\right) = \sum_{j=1}^{K} \phi_j(x) c_j(\mathbf{u}; \theta_j(x)),$$

where $u_i = \mathrm{CDF}_i(y_i|x)$, x is a conditioning variable and $\mathbf{y}$ is a vector of neuronal and/or behavioral recordings. Then, for a data-point $(x, \mathbf{y})$, this probability is equal to:

$$p(\text{clayton}|x, \mathbf{y}) = \frac{p(\mathbf{y}|\text{clayton}, x) \cdot p(\text{clayton}|x)}{p(\mathbf{y}|x)} = \frac{\sum_{j \in \text{Clayton}} \phi_j(x) c_j(\mathbf{u}; \theta_j(x))}{\sum_{j \in \text{All}} \phi_j(x) c_j(\mathbf{u}; \theta_j(x))}.$$

By thresholding $p(\text{clayton}|x, \mathbf{y})$ and $p(\mathbf{y}|x)$, one can select those data-points that constitute the heavy tail of the distribution, which is described by Clayton copulas in our copula mixture model.
