# Supplementary material for: Parametric Copula-GP model for analyzing multidimensional neuronal and behavioral relationships
Source: PLoS Comput Biol. 2022 Jan 28;18(1):e1009799. doi: 10.1371/journal.pcbi.1009799 (PMC8827448; doi:10.1371/journal.pcbi.1009799)
Supplement: Attachment — Submitted filename: rev.pdf [file pcbi.1009799.s005.pdf]

**Manuscript Number:**	PCOMPBIOL-D-21-01071
**Full Title:**	Parametric Copula-GP model for analyzing multidimensional neuronal and behavioral relationships
**Authors:**	Nina Kudryashova, Theoklitos Amvrosiadis, Nathalie Dupuy, Nathalie Rochefort, Arno Onken

In this study, the authors introduce a novel method to estimate correlations among covariates in high-dimensional datasets and improve the quantification of information-theoretic measures. This method combines the use of copulas to characterize complex dependencies among variables and the Gaussian process to describe the smooth variation of stimulation-related or behaviorally-relevant variables over time. The manuscript illustrates in great detail the broad applicability of the method in estimating correlations and information measures in simulations and real datasets. The manuscript is clear and well written. Overall, I believe this method will have a good resonance in the field, therefore I recommend it for publication after addressing the following comments:

Main comments:

1. The authors should provide some background on how the copula mixtures are constructed and the number of parameters involved. Estimating the correlation structure of highly dimensional dataset is a very hard problem (as the authors point out) and the choice of the copula family is crucial. It seems that each copula is identified by a single parameter θ. Is this true at any dimension? Describing high-dimensional data with a single parameter copulas seems restrictive even in mixture models. The authors should also comment on the flexibility of the copulas in describing high dimensional data and the noise distributions.

2. LL112-115: The applicability of the method hinges on the estimation of marginal probabilities. In particular the authors use empirical marginals, however in typical experimental settings the number of trials is not sufficient to sample reliably the neuronal response distributions. When conditioning on time (during a stimulation protocol) or behavioral parameters in in vivo imaging, the number of data points is barely sufficient to estimate mean responses. With our current experimental settings, estimating conditional probabilities from empirical distribution can lead to large errors. The authors should provide a robustness analysis to show how sensitive is their estimate of copula parameters to the unknown marginal distributions.

3. In Sec. 2 the authors apply their method to decouple noise from signal correlation in simulated data from a two-neuron system. In the case where neurons are non trivially coupled by excitory and inhibitory interactions, what is the meaning of the time point of maximal correlation? More generally, are the copula parameters informative about the type of neural circuitry?

4. L167: the author should include in the main text their definition of "confidence intervals".

5. LL204-208: An important source of bias in the estimate of information-theoretic quantities is the sample size used to estimate empirical marginals. Although it's true that MC estimators are unbiased, the authors seem to use a variational inference scheme, which is generally biased. Maybe the authors should clarify.

Minor comments and typos:

1. Typos in the abstract,

   > *[...] However, estimating stimulus or behavioral information encoded in high-dimensional neuronal populations is challenging.*

2. In the abstract,

   > *[...] Moreover, by quantifying the redundancy between neuronal and behavioral variables, our model exposed behaviorally-relevant task structure without providing any explicit cues to the model.*

   I find this sentence a bit obscure.
