# Supplementary material for: Parametric Copula-GP model for analyzing multidimensional neuronal and behavioral relationships
Source: PLoS Comput Biol. 2022 Jan 28;18(1):e1009799. doi: 10.1371/journal.pcbi.1009799 (PMC8827448; doi:10.1371/journal.pcbi.1009799)
Supplement: Attachment — Submitted filename: Authors_responses_CopulaGP.docx [file pcbi.1009799.s006.docx]

**Reviewer #1:** *The manuscript develops the usage of a combination of copulas and Gaussian processes to capture interdependencies in diverse scenarios. The idea is interesting and could be potentially very useful. Additionally, the paper is well written and the technical aspects seem solid. However, I had a hard time to judge the value of the actual contribution, for reasons I explain below.*

We thank the reviewer for the generally positive assessment of our work.

*First, the actual contribution of the paper is not clear. The abstract and introduction implicitly suggest that the idea of combining copulas and Gaussian processes is an original idea of the authors, but then in the result sections it is clarified that actually this has already been developed before in the context of econometric time series data. So, the novelty of the paper seems to be a set of adjustments that allow the translation of these techniques to become suitable to deal with specific types of neural data, but this is not entirely clear. The authors should clarify what are exactly the technical contributions of the paper.*

We agree that the main contributions were not described clearly enough and thank the reviewer for highlighting this point.

While we did have a paragraph on related work about combinations of copulas and GPs in the original introduction (lines 39-43), we have now edited this paragraph to clarify that the combination of copulas and Gaussian processes has been used previously (lines 37-43):

*Here, we use a copula-based approach with explicit conditional dependence for the parameters of the copula model and approximate these latent dependencies with Gaussian Processes (GP). It was previously shown that such a combination of parametric copula models with GP priors outperforms static copula models (Lopez 2013) and even dynamic copula models on many real-world datasets, including weather forecasts, geological data or stock market data (Hernandez 2013). Yet, this Copula-GP approach has never been applied to neuronal recordings before.*

We would also like to point out that we specified our contributions in the paper summary (in layperson terms):

*The novelty of our approach includes 1) explicit modeling of the dependencies; and 2) combining different copulas to describe experimentally observed variability.*

Following the reviewer’s suggestion, we have now also clarified the technical novelty of the paper in more detail in the discussion (lines 625-639):

*The combination of parametric copula models with GP priors has been previously applied to weather forecasts, geological data, or stock market data [Lopez-Paz 2013, Hernandez-Lobato 2013]. Yet, this Copula-GP approach has never been applied to neuronal recordings before. As a distinct feature in neuronal datasets that limited the application of the original method, we observed changes in tail dependencies with regard to the conditioning variable. None of the single parametric copula models could represent such dependencies (Fig. 1). Therefore, the main contributions of our method are the following: 1) we applied gaussian process priors on both the copula parameters and the mixing concentrations in the copula mixture models (see Sec. 5); 2) we have developed the algorithms for constructing such copula mixtures with a Bayesian model selection algorithm (see Sec. 6 and S1 Appx.); 3) we have proposed two approaches for mutual information estimation and validated them against the state-of-the-art information estimators. These contributions made the method flexible enough to describe the dependencies between neurons (Fig. 5C-D and S1 Appx. Fig. A3) and applicable for information-theoretic analysis of neural codes (Fig. 5E-H).*

*As a second point, the paper is focused in a new method to estimate interdependencies, but most of the paper is devoted to the development of examples and little new formal analyses are provided. While the detailed development of these examples may be appreciated by the specialist, in my opinion they may not suffice to establish general & rigorous guarantees that could guide the general usage of these techniques. The authors should complement these examples with more formal analyses, or at least provide strong arguments of why the presented examples are enough to assume that what they illustrate should generalise.*

The examples at the beginning of the paper illustrate the application to neural data and were intended to walk the reader through the interpretation of model components. This part does not claim any novelty on machine learning methodology, but rather puts an abstract (and general) statistical model into neuroscience context.

In later sections of the paper (Sections 3-4), we provide general and rigorous guarantees regarding mutual information estimation (e.g. that the estimator is unbiased given the correct copula parameters and model specification) and validate the model on a variety of datasets. We also refer to the identifiability tests, which are part of our software package. Therefore, we do provide extensive validation for the novel contributions in our paper (namely, for mutual information estimation and identification of copula mixture models).

During revision, we have rewritten the examples section, providing requirements for the types of data that can be analyzed with our model (lines 114-132) and the conclusions that one can draw from it (lines 181-190 and 211-232). We hope that the rewritten section addresses the concerns that were expressed by the reviewer regarding the generalization of our analysis to other types of data.

**Reviewer #2:** *With the increasing complexity of systems neuroscience datasets (both in terms of the number*
*of simultaneously recorded neurons, and in terms of increasing task complexity) there is a*
*pressing need for large scale statistical models that are flexible, yet can be estimated from*
*relatively limited measurements. The copula family of models (which separately models*
*marginal statistics and inter-variable interactions) provides a promising but still relatively*
*unexplored framework for constructing such models.*
*The manuscript integrates copulas with complex structure (copula mixtures for pairwise*
*interactions, smooth dependencies over time in the parameters, and vine copulas for joint*
*statistics) to construct statistical models of stimulus-/time-varying network interactions.*
*Moreover, behavioral variables can be integrated as extra dimensions in the joint model.*
*This framework is applied on simulated data, where it shows sensible reconstruction of*
*underlying data statistics and improved accuracy in mutual information estimates based on the*
*fitted model compared to other state-of-the-art methods. The model is also fitted to a*
*neuroscience dataset from rodents navigating in a VR linear track in order to get a reward.*
*Fitting the model on this data reveals sparse time-varying noise correlations, which seem to*
*reflect the constraints of the task and the low dimensional structure of the associated neural*
*responses.*
*The paper is technically very accomplished, as it brings a range of state of the art machine*
*learning tricks into the statistical toolkit of neuroscience. However, although the presentation of*
*the results generally clear, it does demand a fairly large amount of technical background*
*knowledge from the reader; moreover, I found that the text spends too little time exploring the*
*neuroscientific relevance of the information extracted via the new statistical tool. There are*
*some missed opportunities in engaging with the broader systems neuroscience community,*
*both in terms of explaining what the model is and in terms of educating the reader about how*
*the statistical features extracted by the model can be translated back into neuroscientific*
*knowledge.*
*Some comments and/or suggestions for text improvement :*
*(the order roughly follows that in which the information appears in the text)*
*1. Some more layman introduction into copulas at the beginning of the results could help*
*define the model not just as a mathematical construct but also in analogy with the real*
*world statistical features that a neuroscientist would care about. Motivate the model by the*
*kind of statistical questions that a neuroscientists want to answer — whenever possible.*

We would like to thank the reviewer for the positive assessment of our work and for the detailed and constructive comments. We have added a brief layman introduction to the results section (lines 62-66):

*Many questions in neuroscience require accurate statistical models of neuronal responses to a certain stimulus. For instance, such models can be used for Bayesian decoding of the stimulus [Quiroga, 2009] or for information-theoretic analysis [Timme, 2018] in order to assess the coding precision of a neuronal population. Here we describe a general framework for constructing such statistical models, called Copula-GP; ...*

*2. Line 72: there is an emphasis that the conditioning variable needs to be continuous that I*
*find a bit misleading since KISS-GP and co discretize the variable range*

The statement in line 72 (now 77) is correct: Gaussian Process assumes continuous inputs. The only difference between a full GP and an approximate KISS-GP is that instead of using all available datapoints as inputs, it uses a set of pseudo-inputs. It still assumes a continuous kernel over inputs, so the input variable has to be continuous. The KISS_GP approximation is required for scalability and is justified for the kind of data we have, which has ~20k measured inputs and smooth dynamics that only needs ~100 inputs for accurate estimation. To avoid confusion, we added a clarifying sentence in the Methods section, where KISS-GP is first mentioned (line 744):

*Note that while approximate inference with KISS-GP uses a discrete set of inducing points (pseudo-inputs), it does not imply discretization of the input variable. The Gaussian process still uses a continuous kernel and assumes a continuous input.*

*3. Also: I wonder if it would be useful to add a subscript t for conditioning variables that are*
*time-dependent (within a trial); may help clean up the conditioning on t vs conditioning on x*
*discussion, which I found could have been explained in a cleaner way*

We thank the reviewer for this suggestion. Instead of using subscripts, we explicitly indicate the conditioning variables in brackets (e.g. x(t)) and we follow Bayesian notation (with ‘|’ sign for ‘given’) when talking about conditional variables in probability distributions. Using the subscript might create a false impression that the variable is discretized with respect to time or stimulus, which is not the case (see our response to comment #2 above).

We have revised lines 150-151 and clarified which variables depend on time and/or stimulus:

*Note, that both the neural responses y_i_ and their transformed versions u_i_ still depend on both the stimulus x(t) and time t.*

*4. Line 100: ‘spike inference algorithm’ are usually referred to as ‘deconvolution’ algorithm,*
*here I’d add a concrete reference, for instance the Paninski or similar*

We thank the reviewer for the suggested clarification. We have changed line 106 (previously line 100) accordingly, using the ‘deconvolution’ term and adding the Deneux et al. 2016 reference:

*GLMs are also not directly applicable to calcium imaging data and require some additional spike inference algorithm (deconvolution, e.g. Deneux et al. 2016 [38]) to obtain spikes.*

*5. More general in Results section 2: the assumption that two independent neurons should*
*result into an independent copula, when applied to simulated calcium imaging traces*
*implicitly assumes a factorized ‘measurement’ model, which it’s not clear to me always*
*applies to real calcium imaging data. More generally, the very practically relevant distinction*
*of statistically modeling neural activity directly vs an indirect variable that is correlated to*
*the quantities of interest, here calcium imaging measurements, is swept under the rug a bit*
*too much for my liking.*

Indeed, our analysis of calcium traces implies a factorized ‘measurement’ model (i.e. assumes that individual calcium traces correspond to individual neurons and are not cross-contaminated). We have added a discussion on these assumptions to section 2 (lines 114-132). In this new paragraph, we explained that the raw experimental traces have been decontaminated before we analyzed them with Copula-GP (lines 119 and 405). We should also note that the same independence assumption applies to deconvolution algorithms, which are also typically applied after decontamination (lines 123-125).

*6. Line 114: There is a bit of confusion in the description of the statistical treatment of the*
*marginals. It is advertised as something that ensures maximal flexibility, but not sure that is*
*strictly true: the kernel density estimator makes some simplifying assumptions to get a*
*density from limited data;*

We are grateful to the reviewer for raising this question. We have added a clarification to line 164 (previously 114), which states the limitations of our approach:

“*The conditional marginals p_i(y_i|t) are estimated using the non-parametric fastKDE [Obrien, 2016] algorithm. This approach is suitable for relatively large datasets, which have enough data points for direct estimation of the eCDF (see Sec. 2.5 in S1 Appx.). If the number of datapoints is insufficient, one might consider using parametric marginal models instead. Note that the fastKDE also assumes smooth changes in the marginal distributions with respect to the conditioning variable.”*

Despite the limitations of the marginal estimators, separating marginal statistics from the dependence structure has its benefits. Most importantly, the extracted dependence is insensitive to homeomorphic transformations of the variables, such as scaling. This is a highly desirable property for a model that considers neural and behavioral variables, which are measured in different units (see Table 1 in the new version).

*7. Related: how much data is needed to get the empirical cdf well estimated without any*
*assumptions? This seems particularly important when estimating conditional marginals,*
*since the number of samples is given by the number of repeats of the stimulus, which tends*
*to be pretty restricted in practice…*

We appreciate that the reviewer raised such an important question. Indeed, the number of data points after conditioning on time will correspond to the number of trials, which is often limited. In the experimental data that we were working with, there were 127 trials in total. We hypothesized (and we provide evidence below) that this amount of data is sufficient for estimating empirical marginal distributions, but insufficient for estimating joint distributions of neural responses conditioned on time without further assumptions. Therefore, we opted for a non-parametric estimation of marginal distributions, and parametric copulas for the dependencies.

To show that the number of trials in the experimental dataset is sufficient for estimating the conditional marginals, we performed the following analysis. We first estimated the conditional probability density function p_all_(y|x) for one neuron (e.g. neuron 3, see Fig. 5B) using the fastKDE algorithm and the data from all trials. Next, we consider different subsets of N trials, taken at random (without replacement, 50 independent subsamples), and estimated the conditional pdf p_N_(y|x) again. For each estimate, we compute the distance from an estimated density p_N_(y|x) to the most accurate estimate p_all_(y|x), using Kolmogorov-Smirnov distance and KL divergence (see plots below).

Both measures of the difference between estimated densities decay with the increase of the number of trials and eventually saturate. The Kolmogorov-Smirnov distance is a supremum on a difference between CDFs. Since we use the conditional CDF values for mapping the data on the unit square, the KS distance corresponds to the error in the coordinates of the datapoints in the copula space. If our estimate is inaccurate, the mapping will be incorrect, which will add noise to the datapoints **u**, which we then model with copulas. One can see from the plot above that, on average across shuffles, the maximal error in a CDF drops below 0.1 already for 20 trials (for neuron 3). For subsets with more than 80 trials there were only a few shuffles that had a maximal error greater than 0.1.

We then fixed the number of subsampled trials and repeated the analysis for all of the neurons in the dataset. For the majority of neurons, the maximal KS distance (i.e. the KS distance for the worst-case subsample) was around 0.08 for n=60 and 0.03 for n=120 (n – subsampled trials), and never exceeded 0.14 for n=60 and 0.05 for n=120 (see histograms below). From this analysis, we conclude that the estimation of conditional CDFs is robust and the errors are small (<0.1 in copula space) for the number of trials we have available in the dataset (127).

In applications to other datasets where the number of trials is much smaller (e.g. 10), we suggest finding a suitable class of parametric marginal distributions. However, distinguishing between different shapes of dependence (e.g. Gaussian vs. Clayton) could also become difficult with such scarce data. Therefore, our model is best suited for relatively large datasets (~100 trials or more).

We have added the above analysis to a new Supporting information S1 section 2.5.1.

*8. For the general reader it may important to articulate the distinction between measuring*
*instantaneous dependencies that are time varying vs time-lagged interactions of the kind*
*that a GLM temporal filter would capture. Also there seem to be implicit assumptions built*
*in about the time scale of dependencies that need modeling, which may be helpful to spell*
*out more directly; the coupling terms in a GLM typically imply synaptic interactions*
*between spikes at a ms time scale. What is the time scale of interactions that can be*
*modeled in the copula framework in comparison?*

We have added a suggested clarification regarding the difference between interactions in GLMs and in our model (lines 110-113):

*Our approach is complementary to GLMs: while GLMs consider time-lagged interactions via temporal spike history filters and cannot model instantaneous coactivation [Macke, 2012], our model focuses exclusively on the instantaneous correlations between calcium signals.*

We have also explicitly spelled out the assumptions about the timescales (lines 125-130):

*We will consider the decontaminated recordings y_i(t) to be a surrogate of neural spike trains n_i(t), which can be approximated by blurring (convolving) the spikes with an exponential kernel [Deneux, 2016]. The time constant in this kernel corresponds to calcium decay and also sets the time scale of interactions between spiking neurons, which would be captured by an instantaneous correlation of their calcium traces.*

*9. A comment on the general philosophy of the toy examples as sanity checks that the*
*estimator behaves correctly: in ML one would typically test within model parameter*
*reconstruction first, but this is not quite what is going on here.*

We left the model parameter reconstruction tests out of the main manuscript, but they can be found in Supporting information S1 materials (Section 2, Tables A2-A6).

*While the data is in some*
*sense within the copula model class, the parametrization is very different so it is not at all*
*immediately clear what should one predict about the shape of the extracted copulas in*
*each scenario (especially when the neural responses pass through a not completely*
*specified imaging measurement model)*

Following the reviewer’s suggestions, we have specified the imaging measurement model (see our response to comment #5) and rewrote parts of section 2 in order to explain the shapes of the copulas in more detail (see our response to comment #11 below).

*10. I did not completely understand the depiction of the GLM filters in fig2A, is there a*
*biophysical rationale for that particular choice (more text about the setup of that toy data*
*example would be generally helpful).*

Our intention was to illustrate a case of non-linear dependence (for more details, please see our answer to comment #11).

*11. Missed opportunity: educate the reader about what the shape of a copula tells you about*
*the nature of the interactions between the pair of neurons; the goal is to make those 2d*
*plots intuitive to decipher for a neuroscientist, ideally. Line 160: this dependent is nonlinear*
*comes out of nowhere*

We have added a more thorough explanation of the shape of the dependence, starting from line 173 where we discuss the independence copula:

*The density of points in Fig. 2D illustrates that for every given value of the transformed neural response u ^t^_1_, the conditional distribution of the other neural responses is the same (p(u^t^_2_|u ^t^_1_) =p(u ^t^_2_)∼U[0,1]). This means that the variables u ^t^_1_ and u ^t^_2_ are independent p(u ^t^_1_,u ^t^_2_) =p(u ^t^_2_|u ^t^_1_)p(u ^t^_1_) =p(u ^t^_1_)p(u ^t^_2_), and their copula is the Independence copula.*

We also followed the reviewer’s suggestion and used an opportunity to explain the shape of the nonlinear dependence in lines 211-232 (former line 160):

*This noise correlation model in Fig. 3G can be interpreted as follows. The red point in the middle of each plot in Fig. 3G corresponds to the median response for both neurons: 50% of recorded responses from a given neuron were higher, while 50% were lower. More generally, the values of the transformed neural responses u^t^ correspond to the percentile scores of each response y in a marginal distribution of neural responses. The shades in Fig. 3G correspond to the probability density, i.e. how likely it is to jointly observe a pair of responses u^t^_ex_ and u^t^_inh_. Typically, most of the density is concentrated along one of the diagonals of the unit square: around u^t^_ex_ = u^t^_inh_ if neurons are positively correlated, and around u^t^_ex_ =− u^t^_inh_ if neurons are negatively correlated. In this case, the density is mostly concentrated around the negative diagonal, suggesting that the responses of these two neurons are generally negatively correlated (as expected, given that one of the neurons inhibits the activity of the other). However, this dependence also has a heavy tail: a high degree of association between some of the extreme values of the variables. We can observe this association as a probability mass concentrated in the corner of the unit square (see orange circles in Fig. 3G). It indicates that there is a high probability to observe a strongly activated inhibitory neuron together with the inactivated excitatory neuron. Such asymmetry in the joint trial-to-trial variability of two neurons suggests that their dependence is non-linear: their dependence is not well characterized by a single linear correlation coefficient as there is additional structure in the dependence beyond linear association. In our GLM model, this non-linearity stems from the different timescales of spike history filters: fast inhibitory and slow excitatory neurons.*

*12. Line 171: I am curious about the technical process that allows one to propagate uncertainty*
*in copula parameters into decoding/MI estimates: is that something one can do in*
*analytically or does it rely on sampling from the approx posterior?*

There is no analytical solution for uncertainties in decoding the position/MI estimates, because these quantities involve some integration over GP inputs (i.e. over some combinations of functions modelled by GPs). We clarify this in lines 486-488:

*The confidence intervals (shaded area) were obtained using samples from the Gaussian Process posterior, which reflects the uncertainty in model parameters.*

*13. Line 179: is the fact that the noise correlations are tuned to a particular time point*
*surprising or a ground truth confirmation type of thing? Don't get where that comes from*
*(you only mention something about asymmetric connections after the fact; narratively,*
*would have been better to properly describe the ‘ground truth’ spell out predictions about*
*what should an estimator find and then numerically confirm that it comes out as expected).*

We would like to thank the reviewer for their suggestion for improving the description of our toy examples. As we mentioned above (comments 9-11), we rewrote section 2 in line with what the reviewer proposes. We are now starting by explicitly stating our predictions, and then confirm each prediction with a numerical estimation.2

LL 134-137, Expectation 1: *Since the spiking activity of these neurons in response to a given stimulus is completely independent, we expect that their calcium traces, which are temporally blurred versions of spike trains, to also be independent given the stimulus.*

LL 171-180, Confirmation 1: *The resulting empirical copula correctly demonstrates the absence of dependence between y1(t) and y2(t), as the points are uniformly distributed on a unit square. The density of points in Fig. 2D illustrates that for every given value of the transformed neural response ut1, the conditional distribution of the other neural responses is the same (p(ut2|ut1) =p(ut2)∼U[0,1]). This means that the variables ut1 and ut2 are independent p(ut1,ut2) =p(ut2|ut1) p(ut1) =p(ut1) p(ut2), and their copula is the Independence copula. This result demonstrates that once we made the distinction between noise correlations and stimulus correlations, we could correctly identify that these two neurons are uncoupled (Fig. 2A).*

LL 196-199, Expectation 2: *We expect that these dynamics will be reflected in some non-independent trial-to-trial co-variability of neural responses. Since one neuron inhibits the other, we also expect that their responses will be overall negatively correlated.*

LL 220-224, Confirmation 2: *In this case, the density is mostly concentrated around the negative diagonal, suggesting that the responses of these two neurons are generally negatively correlated (as expected, given that one of the neurons inhibits the activity of the other). However, this dependence also has a heavy tail: a high degree of association between some of the extreme values of the variables.* (see full text in our response to comment #11)

*14. Line 202: would have liked a bit more technical detail on the distinction between the two*
*estimators and why one needs to fit an additional copula model only for one of them.*

In a previous version of the paper, the difference between models has only been explained in the Methods section. Following the reviewer’s suggestion, we have now extended the description of the two models in the Results section and added a reference to the relevant Methods sections with more technical details and equations (lines 268-274):

The first one assumes no conditional dependence in marginals (i.e. p_i_(**y**_i_) =p_i_(**y**_i_|x) for every **y**_i_ and every value of x) and only requires a conditional copula model for p(**y**|x), which is then integrated using the Monte-Carlo (MC) algorithm (over both x and **y**) in order to obtain p(**y**). The second method has no limitations on the conditional dependencies in marginals, but requires an additional copula model for the unconditional distribution p(**y**) apart for a conditional copula model of p(**y**|x) (see Sec. 11 in Methods for details).

*15. In general: MI is one potential quantity of interest but not necessarily the one that most*
*would aim to compute when fitting a joint statistical model to neural responses. One could*
*do a better job in the intro managing expectations and justifying why the main measure of*
*success in this paper is the ability to estimate reasonable (If still biased) MIs.*

We chose mutual information as the main quantity for our model validation, since it is tightly linked to copulas. We mention this relation in the introduction (lines 21-22) and now explain it further at the beginning of section 3 (validation, lines 261-265):

*Static copula models are generally well suited for information estimation, as the mutual information between the variables corresponds to the negative entropy of their copula [Jenison, 2004, Sklar, 1959]. This also applies to the conditional entropy of our conditional copula models H(****y****|x), as it factorizes into marginal and copula components (see equation (11) in Methods).*

*16. More general: it is not clear to me how should an experimentalist looking at a new dataset*
*set out to design hir copula mixture components, especially since the results seem to*
*suggest that it’s really important to get that right (cf line 289)*

We are grateful to the reviewer for pointing out that we did not mention the automatic mixture model construction and model selection procedure anywhere in the introductory sections. We have now added the following sentence to line 93:

*We develop model selection algorithms, based on the fully-Bayesian Watanabe–Akaike information criterion (WAIC), that construct the best suited mixture models for a given data set.*

*17. In the real data: I got really confused about the map between t and x (since self-paced the*
*speed is variable within and across trials so the two are not interchangeable); as far as I*
*understand the conditioning variable ended up being location on track, at a relatively*
*coarse discretization*

The reviewer is correct: the conditioning variable is the location on track, as it is the variable that aligns trials with one another. However, we would like to emphasize that there was no discretization of positions in our method (see our answer to comment #2).

*18. I did not understand what exactly were the behavioral variables that were being jointly fitted*
*with the neural responses, the phrasing in line 347 only added to the confusion about what*
*is being conditioned on and what is being jointly fitted.*

All variables that are jointly fitted were previously listed in Table 3, but the table was buried in the methods section. We have now brought the table into the results section (Table 1). We hope that the table with the list of variables clarifies this point.

*19. I was struggling to find the take home message for the probability density plots in fig 5CD:*
*again, i see challenges in interpretation for these observations. what does it mean for the*
*neural coding properties of the circuit, and is this something expected or independently*
*verified by some other means?*

With figures 5C and 5D we intended to show that we observe some heavy tailed dependencies in the data, both for the noise correlations between two neurons, and for the dependencies between neurons and behavioral variables. Moreover, those dependencies change from one location to another, and can even change from a positive to a negative correlation. The heavy tails in neural noise correlations were expected, based on previous studies that considered various excitatory or inhibitory shared inputs into pairs of neurons [Onken 2009]. Although, such a drastic qualitative change in the dependency in different locations was unexpected.

Following reviewer’s suggestion, we have added the above clarification to the end of section 4.1 (lines 464-471).

*Is there a simpler model that would already capture that kind of structure?*

A simpler model, such as a linear correlation model, would not have captured the tail dependency. We have added the following clarification (line 434-438):

*… it [dependency] was heavy-tailed in some but not all locations (see orange circles in Fig.5C), indicating that the tail-dependence carried information about the location. These heavy-tailed dependencies can be captured by our copula models but would have been missed if the noise correlations were only characterized with a linear (Pearson) correlation coefficient.*

*20. I also did not understand what were the variables between which MI was computed in line*
*407, which made the increase of MI in the reward zone also difficult to map into neural*
*coding properties of potential interest*

We have added a clarification to that sentence, specifying the pairs of variables (lines 488-491):

*For both examples, the MI between variables (between two neurons in Fig. 5E and a neuron vs. the licking rate in Fig. 5F) peaks in the reward zone located at 120-140 cm in this task (Fig. 5A).*

With these two examples, we aimed to show the generality of our approach, that it is applicable for modelling noise correlations between neurons and for conditioning neuronal responses on behavioral variables.

*21. It would help if you could add intuitive explanation of how a vine copula works and what*
*those parameters map into, at the minimal level required to understand the results in sec*
*4.3;*

Following the reviewer’s suggestion, we have added a brief introduction into vine copulas to section 4.3:

*Finally, we constructed a C-vine model describing the distribution between all neuronal and behavioral variables in the dataset ({ux1...ux109}, dimux= 109, see Table 1). The C-vine model decomposes the high-dimensional distribution into a hierarchy of bivariate copulas, where each level of the hierarchy (a vine tree) adds a conditional dependence on one of the variables. Such factorization allows one to escape the ‘curse of dimensionality’ while still accounting for the higher-order correlations between the variables [Onken 2016].*

*this is just a more expanded vs of the question what does jointly modeling the*
*behavioral variables teaches us abut the coding properties of the recorded neurons*

We have clarified the take home message from Fig. 5E-F in the following way (lines 511-514):

*In those regions where the redundancy was high, a larger portion of neural variability can be accounted for, based on the activity of the other neurons and on the current behavior of the animal.*

*22-3. Maybe I missed it but the comparison to other methods makes statements about sample*
*complexity of estimator (how much data needed, e.g. line 481) that I did not see any direct*
*support in the results for the statement that other joint models cannot do conditionals, stimulus dependencies etc is simply not true. There exists at least one stimulus dependent ising model (from Tkacik and co), and many latent dynamical systems models of joint activity include explicit stimulus*
*dependencies either in the observation model (e.g. PLDS by Macke) or in the latent space*
*(e.g. recent work from Scott Linderman)*

The word ‘flexible’ in our text was misleading. Therefore, we have clarified the model comparison section (lines 567-581), explaining in which way our model is similar to the conditional maximum entropy model [Tkacik 2013, Gardella 2016, O’Donnell 2017] and in which ways it is, nonetheless, more `flexible’ (i.e. represents more complex pairwise conditional dependencies, if needed):

*Some of these maximum entropy models utilized conditioning on the overall population activity in order to take some of the higher order correlations into account [Tkacik 2013, Gardella 2016, O’Donnell 2017]. These models resemble the conditioning used in the first tree of our vine copulas, especially when we select the global neuropil activity as the first conditioning variable (see Sec. 7). However, all these population tracking maximum entropy models assumed linear coupling of each individual neuron with the population rate, characterized by a single parameter (thus,∼N^2^ parameters in total). We relax this constraint by introducing mixtures of copulas from different copula families, which model complex nonlinear pairwise dependencies between variables. The complexity of the copula mixture ‘adapts’ to the complexity of the data, since we select the least complex model that can capture the dependence via the information criterion (WAIC). As a result, our semi-parametric Copula-GP model takes the best of both worlds, performing well given a limited number of high-dimensional samples, and, at the same time, being more flexible in representing the conditional dependencies than maximum entropy models.*

*24. Methods: automatizing hyperparameter optimization seems rather important to make the*
*tool usable by other on new datasets*

We absolutely agree with the reviewer, and we have performed this optimization, as described in Section 5, subsection on Approximate inference (lines 748-750):

*Equation [ELBO] enables joint optimization of the GP hyperparameters (constant mean and two kernel parameters: scale and bandwidth) and parameters of the variational distribution q (mean and covariance at the inducing points) [Hensman 2015].*

*25. Methods: can one and maybe should one regularize the components of the mixture in*
*some way (thinking sparse mixtures may be a bit more interpretable, but then maybe not)*

We do regularize the components of the mixtures by means of the Watanabe–Akaike information criterion (WAIC). Simple mixtures are preferred over complex ones following the WAIC, which we use for mixture model selection. The WAIC balances the posterior predictive density vs. the complexity of the model. We can imagine that one might modify the WAIC equation and increase the weight of the complexity term in order to shift the balance towards a simpler model. Although, such an approach would be less principled than the selection based on a proper WAIC.

*26. Big picture: it is at the very list unusual when modeling neural activity simultaneously with*
*stimuli and/or behavior that one conditions on the stimulus, but not the behavioral outcome*
*(theoretical frameworks usually talk about encoding or decoding models of circuit*
*computation, and this is neither). I think more justification is necessary about the practical*
*use of such a joint model in interpreting what the circuit computes in relation to the*
*stimulus and behavior.*

We assume that our Copula-GP model is conditioned on a continuous variable that determines the phase of the trial, which, depending on the task, could be time, or space, or orientation, etc. Conditioning on this variable allows us to separate within-trial vs. across-trial variability. If all trials had the same sequences of stimuli x(t), then this is also interpreted as a separation between noise vs. stimulus correlations, as we mentioned in the discussion (line 610):

*If this variable also uniquely determines the stimulus, then the components of the model (2) can be interpreted in terms of ‘noise correlations’, approximated by a parametric copula.*

We have now adjusted the description of the conditional model in a toy example to clarify the choice of the conditioning variable (line 164):

*The conditional marginals pi(yi|t) in this model account for the within-trial variability (e.g. dynamics of responses), while the copula model accounts for the trial-to-trial variability in neural responses.*

*27. Ultimately, having a great statistical model of the data is not all that counts when it comes*
*to basic science; some may argue (and have argued) that a simpler interpretable model*
*that captures reasonably the data is in some sense better than a complex yet*
*uninterpretable model with a better quantitative fit. For the tool to be widely used, it needs*
*not only to do well in ML terms but to extract scientific knowledge — this is the point were*
*the argument fails short as presented at the moment. At the very least, one should provide*
*clearer guidelines on what kind of questions is the framework more or less suitable for,*
*within the space of joint statistical descriptions of neural responses during behavior.*

As a statistical model, Copula-GP indeed has a limited mechanistic interpretability. Nonetheless, unlike other statistical models, it makes the dependence structures explicit and provides intuitive visualizations of the shape of the dependence, which we hopefully describe more clearly, following the suggestions in comment #11 above.

We have added more emphasis on what our model can and cannot do throughout the text (lines 181-186 and lines 241-257). Briefly, we warn the reader against the naïve mechanistic interpretation of noise correlations, but emphasize the usefulness of an accurate statistical model for Bayesian decoding or information-theoretic analysis. The latter is the task that is best suited for copulas, since their negative entropy corresponds to the generalized redundancy between variables.

*Overall, I think this is a pretty good paper already and my criticism is meant to make it better*
*by increasing the long term impact of the tool on the community. Not everything needs to*
*necessarily be addressed for the manuscript to be worthy of publication, but I do think*
*addressing as many as possible will make the paper ultimately stronger.*

We thank the reviewer again for the constructive and useful comments which helped us to strongly improve the manuscript.

***Reviewer #3:*** *In this study, the authors introduce a novel method to estimate correlations among covariates in high dimensional datasets and improve the quantification of information-theoretic measures. This method combines the use of copulas to characterize complex dependencies among variables and the Gaussian process to describe the smooth variation of stimulation-related or behaviorally-relevant variables over time. The manuscript illustrates in great detail the broad applicability of the method in estimating correlations and information measures in simulations and real datasets. The manuscript is clear and well written. Overall, I believe this method will have a good resonance in the field, therefore I recommend it for publication after addressing the following comments:*

We would like to thank the reviewer for a thorough review and useful comments.

***Main comments:***

*1. The authors should provide some background on how the copula mixtures are constructed and the number of parameters involved. Estimating the correlation structure of highly dimensional dataset is a very hard problem (as the authors point out) and the choice of the copula family is crucial. It seems that each copula is identified by a single parameter θ. Is this true at any dimension? Describing high-dimensional data with a single parameter copulas seems restrictive even in mixture models. The authors should also comment on the flexibility of the copulas in describing high dimensional data and the noise distributions.*

In the initial version of the manuscript, we did not explicitly mention the total number of parameters in the text, and only implicitly used the number in section 8 on algorithmic complexity. We have corrected this omission and added a paragraph to the end of section 7 (Copula vines).

Briefly, if we only used single parameter bivariate copulas, then the total number of parameters for an N-dimensional vine copula model would have been N(N-1)/2. However, we are using copula mixture models with M elements, which have max(0,2M-1) parameters. Mixtures can have 0 parameters if variables are independent. Therefore, the total number of parameters in the model strongly depends on the data. For neural data, many pairs of variables are conditionally independent, so many bivariate copulas are Independence copulas (with 0 parameters). This drastically reduces the number of parameters, which we discuss in Section 8 on algorithmic complexity.

*2. LL112-115: The applicability of the method hinges on the estimation of marginal probabilities. In particular the authors use empirical marginals, however in typical experimental settings the number of trials is not sufficient to sample reliably the neuronal response distributions.*

We fully agree with the reviewer that our approach requires extra justification, which we provided above in an answer to Reviewer 2 (point 7). Briefly, we show that the number of trials in our data (n=127) was sufficient for empirical estimation. We also added a suggestion to the text (lines 166-168), indicating that in an alternative case where data is scarce, one could use parametric marginal models.

*When conditioning on time (during a stimulation protocol) or behavioral parameters in in vivo imaging, the number of data points is barely sufficient to estimate mean responses. With our current experimental settings, estimating conditional probabilities from empirical distribution can lead to large errors. The authors should provide a robustness analysis to show how sensitive is their estimate of copula parameters to the unknown marginal distributions.*

The authors would like to thank the reviewer for raising this important question. Indeed, the number of data points after conditioning on time will correspond to the number of trials, which is often limited. In our response to reviewer 2 (point 7), we provide an additional analysis illustrating that the estimation of condition CDFs is robust and the errors are small (<0.1 in copula space) for a given number of trials (127). The second part of the question refers to the sensitivity of the estimated copula parameters to the noise that comes from erroneous estimates of the marginals. To answer this question systematically, we used the synthetic data from Figure 3, which is well described by a single copula. We have perturbed every CDF estimate at a random location x_p_, shifting the CDF value up or down by a fixed step ε. The rest of the function: CDF(x; x>x_p_) for ‘up’ perturbation or CDF(x; x<x_p_) for down perturbation, was scaled linearly such that the CDF values remain within the range [0,1].

We have then varied the magnitude of a fixed step ε and measured the error in the copula parameter estimate. We have observed that for ε<=0.05 (e.g. ε=0.05 or ε=0.02 in the figure), most of the random perturbations do not affect the estimate of the copula parameter. This magnitude of the perturbation (ε) can be related back to the Kolmogorov-Smirnov distance between estimated CDF based on subsampled trials (see our response to reviewer 2, point 7). Given the amount of data we have (127 trials), the error in CDF estimates is well below 0.05 level.

We have added the above analysis to a new Supporting information S1 section 2.5.2.

*3. In Sec. 2 the authors apply their method to decouple noise from signal correlation in simulated data from a two-neuron system. In the case where neurons are non trivially coupled by excitory and inhibitory interactions, what is the meaning of the time point of maximal correlation? More generally, are the copula parameters informative about the type of neural circuitry?*

As a statistical model, Copula-GP does not provide a mechanistic description of the neural circuits. In a particular toy example, the point of maximal correlation also corresponds to the maximal mutual information between two neurons. It indicates that their responses become highly redundant, but without knowing the circuit, we would not have been able to tell whether this redundancy comes from the recurrent activity or from the shared input into neurons.

Therefore, due to a number of questions from the reviewers regarding mechanistic interpretations, we have rewritten the conclusions of section 2 in order to explicitly state what our model can and cannot do (lines 246-257):

*The main advantage of using copula models [Jenison, 2004] is their expressive power in modeling the shape of the relationship between variables, which, in this case, is asymmetric due to the dynamic connections in the neuronal circuit being asymmetric (Fig. 3). It was also shown previously that shared excitatory or inhibitory inputs can result in non-trivial (non-Gaussian) dependencies between neurons [Onken, 2009]. While copula models of the noise correlations do not provide any mechanistic interpretation of these dependencies (e.g. whether neurons are recurrently coupled or share an input), their expressive power allows one to build statistical models for information-theoretic analysis or Bayesian decoding. An additional explicit fully-Bayesian parameterization provided by a GP (Fig. 3F) extends the copula model to time-dependent, location-dependent or otherwise continuously parameterized behavioral tasks.*

*4. L167: the author should include in the main text their definition of “confidence intervals”.*

We have added a definition in parenthesis (lines 233-234):

*The confidence intervals (95% CI, two standard deviations) shown in Fig.3F correspond to the uncertainty in model parameters, captured by the Gaussian process.*

*5. LL204-208: An important source of bias in the estimate of information-theoretic quantities is the sample size used to estimate empirical marginals. Although it’s true that MC estimators are unbiased, the authors seem to use a variational inference scheme, which is generally biased. Maybe the authors should clarify.*

We would like to thank the reviewer for this useful suggestion. We have now clarified the factors that can affect the accuracy of the model fit and thereby skew the information estimate (lines 276-278):

*The only source of bias in our information estimates comes from the accuracy of the model fit obtained via variational inference, which is inevitably biased.*

We agree that the quality of empirical marginal estimation can potentially contribute to the biases in information estimation. If the true copula parameters are known, the rest of the estimation is unbiased, as the reviewer also acknowledged.

***Minor comments and typos:***

*1. Typos in the abstract, [...] However, estimating stimulus or behavioral information encoded in high-dimensional neuronal populations is challenging.*

We have corrected the sentence:

*However, estimating stimulus or behavioral information* ***that is*** *encoded in high-dimensional neuronal populations is challenging.*

*2. In the abstract, [...] Moreover, by quantifying the redundancy between neuronal and behavioral variables, our model exposed behaviorally-relevant task structure without providing any explicit cues to the model. I find this sentence a bit obscure.*

We have clarified this sentence:

*Moreover, by quantifying the redundancy between neuronal and behavioral variables, our model exposed the location of the reward zone in an unsupervised manner (i.e., without using any explicit cues about the task structure).*
